# Targeted HIV testing modalities in Zimbabwe: Achieving effectiveness through a revised model

**DOI:** 10.1016/j.gloepi.2026.100288

**Published:** 2026-09-06

**Authors:** Hamufare Dumisani Mugauri, Owen Mugurungi, Joconiah Chirenda, Kudakwashe Takarinda, Mufuta Tshimanga

**Affiliations:** a The University of Zimbabwe, Global, Public Health and Family Medicine Department, Harare, Zimbabwe; b Ministry of Health and Childcare, AIDS and TB Unit, Harare, Zimbabwe; c Organisation for Public Health Interventions and Development (OPHID), Harare, Zimbabwe

**Keywords:** HIV testing, Risk screening, Index testing, Provider-initiated testing, HIV self-testing, Linkage, Zimbabwe

## Abstract

**Introduction:**

HIV testing remains central to epidemic control, but awareness of HIV status in Zimbabwe remains below UNAIDS targets. We evaluated a targeted HIV testing model using a risk-screening tool to triage clients for same-day provider-initiated HIV testing, while routing screen-negative clients to HIV self-testing.

**Methods:**

We conducted a mixed-methods implementation evaluation across eight provinces from April 2021 to June 2022. Thirteen candidate screening questions were evaluated in 7825 clients and reduced to five domains for assessment of the final combined rule in 2116 complete-case assessment records. The rule screened clients last tested at least 3 months previously, or never tested, who reported at least one additional risk or symptom criterion. We also conducted 20 healthcare-worker interviews and reconstructed the index-testing cascade for 6308 index cases.

**Results:**

The final combined rule achieved 94.1% sensitivity, 19.5% specificity, 9.2% positive predictive value, 97.4% negative predictive value, 18.0% testing volume reduction, and a number needed to test of 11. In the index-testing cascade, 66.4% of index cases were offered partner testing; 5080 contacts were elicited, 78.6% were tested, 43.5% tested HIV positive, and 91.8% initiated antiretroviral therapy. Positivity was associated with anonymous tracing (aOR 8.46, 95% CI 3.37–22.75), ≤7-day testing (2.78, 2.44–3.18), male sex (3.09, 2.74–3.49), and first-time testing (1.65, 1.43–1.91).

**Conclusion:**

The targeted model maintained high screening sensitivity while modestly reducing same-day provider-initiated testing volume, and index testing achieved high positivity and ART linkage among tested contacts. The screening rule should be interpreted as a triage mechanism rather than a diagnostic discriminator, while implementation of the broader model requires timely contact tracing, differentiated testing options, and reliable linkage systems.

## Introduction

HIV testing remains central to epidemic control, yet an estimated 8.1 million people worldwide are unaware of their status [1], [2]. In sub-Saharan Africa, where over half of all people living with HIV reside, precision-based testing is critical to maintain yield and conserve resources [3]. Zimbabwe has progressed toward the UNAIDS 95–95–95 targets: 97.0% of diagnosed people are on antiretroviral therapy, and 90.3% achieve viral suppression by 2020; however, only 86.8% know their status [4]. This gap creates a need for differentiated HIV testing approaches that preserve case detection while improving testing efficiency [5]. Index testing can deliver high yields but has faced implementation and fidelity challenges in Zimbabwe, leading to missed case-finding opportunities [[6], [7]]. Evidence from Zimbabwe and the wider Southern African region suggests that risk screening, index testing, and differentiated testing modalities can improve HIV testing efficiency, but their effectiveness depends on implementation fidelity, clear eligibility criteria, and integration within routine service-delivery workflows [8].

To address these inefficiencies, we evaluated Zimbabwe's targeted HIV testing model as an integrated programme approach combining risk screening, index testing, and HIV self-testing pathways. Risk screening is applied when clients present for routine services and is intended to identify those who should receive same-day provider-initiated HIV testing, while clients who screen negative may be routed to HIV self-testing or scheduled/repeat testing according to programme procedures. The final operational HIV risk-screening tool is provided in Appendix A, while the 13 candidate screening questions evaluated during tool development and the five domains retained in the final tool are presented in Appendix *B. index* testing operates later in the pathway, after an HIV-positive client has been identified, and aims to reach sexual partners and biological children who may have undiagnosed HIV. These modalities are evaluated together because they are complementary components of the same targeted-testing model: screening improves triage for routine testing encounters, index testing increases case-finding among contacts of HIV-positive clients, and HIV self-testing provides an additional client-centred pathway for people not immediately tested through facility-based services. Our objectives were to: (1) evaluate the performance of a brief HIV risk-screening rule for identifying clients with confirmed HIV-positive test results among those presenting for HIV testing services; (2) reconstruct the index-testing cascade and examine factors associated with HIV positivity among elicited contacts; (3) describe healthcare-worker perspectives on implementation barriers and enablers affecting risk screening and index testing; and (4) assess programme fidelity and use the findings to refine the national targeted HIV testing model.

## Methods

### Study design

We conducted a mixed-methods implementation evaluation comprising four linked components: (i) quantitative evaluation of candidate HIV risk-screening items and assessment of the final combined screening rule, (ii) quantitative reconstruction of the index-testing cascade and analysis of factors associated with contact HIV positivity, (iii) qualitative in-depth interviews with healthcare workers to explore implementation enablers and barriers, and (iv) a programme fidelity assessment to identify delivery gaps relevant to the national targeted HIV testing model. Findings from these components were integrated during interpretation to inform refinement of Zimbabwe's targeted HIV testing approach. This framing distinguishes the study from a purely diagnostic validation study and reflects the programme implementation context in which the screening rule, index testing, HIV self-testing, and fidelity supports were evaluated together.

### Study setting and period

The evaluation was conducted across eight provinces in Zimbabwe: Harare, Manicaland, Mashonaland East, Mashonaland Central, Mashonaland West, Masvingo, Midlands, and Matabeleland South, between April 2021 and June 2022. Quantitative data sources included public primary, secondary, and tertiary facilities, together with selected community outreach points, to capture variation in HIV burden, service-delivery platforms, and operational conditions across settings. For the screening-tool component, the initial item-evaluation phase was conducted from 1 to 30 April 2021, and assessment of the final combined screening rule was conducted from 4 to 15 October 2021. Qualitative interviews were conducted from 15 November to 10 December 2021 in selected facilities in Harare and Mashonaland East to explore healthcare worker perspectives on screening and index-testing implementation.

### Sampling strategy and participants

#### Province and facility selection

Provinces were selected purposively to reflect variation in HIV burden, urban-rural distribution, service-delivery context, and programme implementation capacity. Within provinces, public facilities were selected to include different levels of care, higher- and lower-volume HIV testing settings, and facilities with routine implementation of targeted testing activities. Facilities were purposively selected to represent both higher- and lower-volume HIV testing services (HTS) settings and to align with programme implementation capacity. For the screening-tool component, 64 facilities contributed to the initial item-evaluation phase and 35 facilities contributed to assessment of the final combined screening rule. The 35 assessment facilities were a subset of the facilities participating in the evaluation phase. The index-testing cascade analysis used routine programme data from 50 facilities in Harare Urban and Matabeleland South.

#### HTS clients for screening-item evaluation and assessment of the final combined rule

For the screening-tool component, healthcare workers approached consecutive clients aged ≥15 years who were seeking HIV testing services during routine service hours. Inclusion criteria were age ≥ 15 years, ability to provide informed consent or assent, and not being previously known to be HIV-positive. Exclusion criteria were acute illness requiring urgent care, repeat testing within <3 months, and inability to consent. The initial item-evaluation phase enrolled 7825 clients. The assessment of the final combined screening rule enrolled 2140 clients. The 24 excluded records had incomplete screening data and/or non-definitive HIV test results.

#### Index clients and elicited contacts

For the index-testing component, routine programme data were abstracted for index clients diagnosed or newly enrolled during the study period and for their elicited contacts. Index-client inclusion criteria were newly diagnosed or newly enrolled HIV-positive clients with documented index-testing offer. Contact inclusion criteria were elicited sexual partners or biological children with recorded testing outcomes. After de-duplication, 6308 index clients and 5080 contacts were included in the cascade and regression analyses.

#### Data sources, extraction and quality assurance

Quantitative data were abstracted from facility HIV testing services (HTS) registers, index-testing registers, and District Health Information Software 2 (DHIS2) exports using a standardized extraction tool. Two trained abstractors extracted data independently at each site, with real-time cross-checks and weekly supervisory review to improve completeness and consistency.

De-duplication was undertaken in EpiData using composite identifiers, including combinations of name, sex, age or date of birth, facility, and test date and, where available, national identification number or ART number. Potential duplicates and internal inconsistencies were reviewed manually before finalizing analytic datasets.

For the screening-tool component, complete-case analyses were used for performance estimation. The record-level assessment dataset contained facility identifiers, service-entry point, sex, age, prior HIV testing history, item-level screening responses, and the current HIV test result, allowing screening responses to be linked to definitive HIV outcomes. Of the 2140 clients enrolled in the assessment of the final combined screening rule phase, 24 were excluded from complete-case analyses because of incomplete data and/or non-definitive HIV test results, leaving 2116 records for assessment analyses.

For the index-testing component, index–contact pairs were de-duplicated before cascade reconstruction and regression modelling. For the province-level programme-output component, aggregate reports were available for Manicaland and Matabeleland South. These reports contained index-testing and HIV self-testing indicators used for descriptive reporting in Table 4.

#### Screening tool development, implementation and assessment of the final combined rule

*Tool development and item selection:* The HIV risk-screening tool was developed by the Ministry of Health and Child Care in collaboration with a supporting-partners study group, comprising programme personnel and supporting partners. Development drew on Zimbabwean programme experience, published evidence, and HIV risk-profiling questions previously used locally and within the region. An initial set of 13 candidate screening questions was assessed during routine HIV testing service delivery among 7825 clients across 64 facilities. The complete set of candidate questions is provided in Appendix B. Each question was evaluated against the client's definitive HIV test result. Item retention was informed by screening performance and operational feasibility, with the objective of identifying a parsimonious set of domains that could maintain high sensitivity while remaining practical for routine service delivery. Five domains were retained: self-perceived HIV risk, whether a sexual partner had tested HIV-positive in the previous 2 years, timing of the last HIV test, poor health, and symptoms or signs of a sexually transmitted infection.

*Tool content, implementation and decision criteria:* The wording of some retained domains differed between the initial item-evaluation phase and the subsequent assessment of the final combined rule. During the initial item-evaluation phase, the candidate questions included self-perceived HIV risk; having a sexual partner who had tested HIV-positive in the previous 2 years; having last tested for HIV more than 1 year previously or never having tested; poor health in the previous 12 months; and symptoms or signs of a sexually transmitted infection in the previous 12 months. The full set of 13 candidate questions, with the five retained domains identified, is provided in Appendix B.

Before subsequent field assessment, the implementation rule was refined to standardize provider interpretation and align the tool with routine HIV testing procedures. Under the final combined rule, clients were screened in for same-day provider-initiated HIV testing if they had last tested for HIV at least 3 months previously, or had never tested, and reported at least one additional risk or symptom criterion. The recall periods for poor health and symptoms or signs of a sexually transmitted infection were shortened from 12 months to 3 months to reduce recall ambiguity and align the questions with the final implementation procedure. The final operational risk-screening tool, including response options and the screening decision fields, is reproduced in Appendix A.

Because the wording, recall periods, and testing-interval criterion changed between phases, the October 2021 phase should be interpreted as an assessment of the final combined implementation rule rather than as validation of an unchanged instrument. Comparisons between the initial item-evaluation phase and the subsequent assessment phase are therefore interpreted as programme-development evidence. Further external validation of the final combined rule is required to establish its performance and transportability in other populations and service-delivery settings.

Healthcare workers administered the final screening tool before HIV testing during routine service encounters. Clients who met the final combined screening rule were prioritized for same-day provider-initiated HIV testing. Clients who did not meet the rule were not categorically excluded from HIV testing; depending on programme procedures, client preference, individual circumstances, and service-delivery capacity, they could be offered HIV self-testing or advised to undergo scheduled or repeat testing. The final operational tool and its decision fields are provided in Appendix A.

*Performance measures:* Screening-rule performance was assessed against the definitive HIV test result among clients with complete screening responses and conclusive HIV test outcomes. We calculated sensitivity, specificity, positive predictive value, negative predictive value, testing-volume reduction, and number needed to test, with 95% confidence intervals where applicable. Sensitivity was defined as the proportion of HIV-positive clients classified as screen-in, and specificity as the proportion of HIV-negative clients classified as screen-negative.

Positive predictive value was the proportion of screen-in clients who tested HIV-positive, and negative predictive value was the proportion of screen-negative clients who tested HIV-negative. Testing-volume reduction was defined as the proportion of clients classified as screen-negative under the final combined rule, and number needed to test was defined as the number of clients tested to identify one HIV-positive person. Raw contingency-table counts are reported to support reproducibility of the performance estimates.

#### Index testing cascade construction and analysis

The index-testing cascade was reconstructed using routine facility index-testing registers and District Health Information Software 2 (DHIS2) summaries. Cascade stages included documentation of index-testing offer, acceptance, contact elicitation, contact follow-up, contact testing and receipt of results, HIV positivity, initiation of antiretroviral therapy among contacts testing HIV-positive, and linkage to prevention services among contacts testing HIV-negative. Percentages were calculated using the relevant denominator at each cascade step, and denominators are stated in the Results, tables, and figure captions. These routine programme data were used to quantify attrition across the cascade and to identify programme points at which yield or linkage could be strengthened.

HIV self-testing was included as a differentiated testing pathway within the targeted testing model. In the index-testing context, HIV self-testing could be used to extend testing access to eligible contacts who were not immediately tested through facility-based or provider-assisted approaches, with reactive self-test results requiring confirmatory testing according to national procedures. HIV self-testing was also relevant for clients who screened negative for same-day provider-initiated testing but remained eligible for alternative testing pathways. HIV self-testing outputs were therefore interpreted as complementary programme outputs rather than as direct measures of facility-based index testing alone.

For province-level programme outputs, aggregate programme reports were available for selected provinces and included numbers of index cases offered testing, contacts elicited, contacts tested, contacts testing HIV-positive, linkage outcomes, and HIV self-testing outputs. Where percentages and numerators were reported in aggregate programme summaries, denominators were derived arithmetically from the documented values and are presented transparently in the relevant table footnotes. These derived denominators were used only for descriptive programme-output reporting and were not used to generate individual-level screening performance estimates or regression models.

To examine factors associated with HIV positivity among elicited contacts, the contact was used as the unit of analysis. Logistic regression was used to estimate crude and adjusted odds ratios with 95% confidence intervals. Covariates were selected a priori based on programme relevance, published evidence, and availability in routine programme data, and included sex, relationship to the index client, tracing modality, time from elicitation to testing (≤7 days versus >7 days), and prior HIV testing history. Because HIV positivity among tested contacts was common, odds ratios are interpreted as measures of association rather than approximations of risk ratios, and this limitation is considered in the Discussion.

#### Quantitative statistical analysis

For the screening-tool component, we estimated sensitivity, specificity, positive predictive value (PPV), negative predictive value (NPV), testing-volume reduction, and number needed to test (NNT), with 95% confidence intervals where applicable. Individual-item performance estimates were derived from the initial item-evaluation dataset, whereas performance of the final combined screening rule was derived from the complete-case assessment dataset. Testing-volume reduction was defined as the proportion of clients screened out from same-day provider-initiated HIV testing relative to all clients presenting for HIV testing services under the screening criteria, and NNT was defined as the number of clients who would need to be tested to identify one HIV-positive individual. To improve reproducibility, the revised manuscript reports the raw contingency-table counts underlying diagnostic estimates in the main tables and supplementary material.

For the assessment of the final combined screening rule, complete-case analyses were conducted using records with complete screening responses and definitive HIV test outcomes. Of the 2140 clients enrolled in the assessment of the final combined screening rule phase, 24 were excluded from complete-case analyses because of incomplete data and/or non-definitive HIV test results, leaving 2116 records for assessment analyses. Prevalence in the complete-case assessment dataset was therefore calculated using 2116 as the analytic denominator.

For the index-testing strand, the contact was the unit of analysis for modelling factors associated with HIV positivity. Crude associations between each factor and HIV positivity were first examined using category-specific HIV-positive and HIV-negative frequencies, and crude odds ratios were calculated from these counts. Multivariable logistic regression was then used to estimate adjusted odds ratios with 95% confidence intervals for prespecified covariates, including sex, relationship to the index client, tracing modality, time from elicitation to testing, and prior HIV testing history. The regression table reports both crude and adjusted estimates together with the frequencies needed to derive the crude measures of association.

Model assumptions for the multivariable logistic regression were assessed using standard diagnostic procedures, including checks for influential observations and multicollinearity using variance inflation factors. Analyses were conducted in Stata version 12.1, and de-duplication and preliminary data processing were completed in EpiData. Where crude 2 × 2 frequencies differed slightly across factors because of variable-specific data availability in the analytical dataset, factor-specific denominators were retained rather than imposing a uniform denominator across all rows.

#### Qualitative methods

*Healthcare-worker sampling*: For the qualitative component, we used purposive maximum-variation sampling to recruit healthcare workers involved in HIV risk screening and/or index testing. Interviews were conducted in selected facilities in Harare and Mashonaland East. These provinces were selected to capture variation in implementation context, including high-volume urban service delivery and mixed urban-rural facility settings where targeted testing activities were being implemented. Eligible participants included primary counsellors, registered/general nurses, and sisters-in-charge or matrons who had been involved in screening and/or index testing during the study period, had worked at the facility for at least 6 months, and were willing to provide written informed consent. Recruitment aimed to include variation by cadre, facility level, and involvement in screening or index-testing activities.

*Data collection:* Semi-structured in-depth interviews were conducted from 15 November to 10 December 2021 using an interview guide covering screening-tool usability, workflow integration, index-testing implementation, use of standard operating procedures and job aids, staffing and training, documentation and data tools, and perceived implementation challenges and solutions. Interviews were conducted by 4 trained interviewers with backgrounds in public health, who were not direct supervisors of the participating healthcare workers. All interviews were conducted in English in private spaces at participating facilities. Written informed consent was obtained before each interview. Interviews were audio-recorded with consent and supplemented by field notes. The interview guide was developed to elicit provider perspectives on how risk screening and index testing were implemented in routine settings, including factors affecting consistency, feasibility, fidelity, confidentiality, contact elicitation, tracing, and follow-up. A blank version of the interview guide is provided as supplementary material.

*Transcription:* Audio recordings were transcribed verbatim by trained members of the study team. Study team members checked the transcripts against the audio recordings and field notes to assess accuracy, completeness, and preservation of participants' intended meaning. Transcripts were de-identified before coding and analysis.

*Analysis and rigour:* Transcripts were analyzed thematically in NVivo 10 using a combination of deductive and inductive coding. An initial codebook was developed from the study objectives, interview guide, and early review of transcripts, then refined iteratively to accommodate emergent themes. Coding was conducted by trained members of the study team with experience in public health and HIV programming. Coding decisions and emerging themes were discussed within the team, and differences were resolved by consensus. Interviewers contributed to analysis through field debriefs and interpretation of contextual observations but did not independently determine final themes unless they were part of the coding team. Where more than one analyst coded transcripts, coding differences were discussed and resolved by consensus.

Saturation was assessed using two complementary processes. During data collection, interviewers completed field debriefs and reviewed interview summaries to identify whether new implementation issues were still emerging. After transcription, saturation was confirmed during coding when no substantively new codes or themes emerged in the final interviews. After coding, coded excerpts were summarized in analytic matrices to compare themes across cadre groups, facility types, and involvement in risk screening or index-testing activities. Thematic findings were then interpreted alongside screening performance, index-testing cascade results, and programme fidelity findings to identify implementation factors influencing delivery of the targeted testing model.

#### Programme fidelity assessment

Programme fidelity was assessed using a logical-framework checklist structured around targeted-testing inputs, processes, and outputs across participating districts. Fidelity domains included availability and use of standard operating procedures and job aids, staffing, training, screening tools, HIV self-testing commodities, index-testing documentation tools, routine data recording, and evidence of linkage or referral processes.

Fidelity data were collected by trained programme assessment teams using standardized checklists. Information was obtained through direct review of facility registers, screening tools, index-testing registers, HIV self-testing stock or distribution records, training records, supervisory reports, and available standard operating procedures or job aids. Where staff reports were used to clarify implementation practices, they were cross-checked against available records or direct evidence where possible. Self-reported information alone was not treated as sufficient evidence for scoring unless supported by documentation, observed tools, or routine programme records.

Each fidelity item was scored as present (=1) or absent (=0), and items were equally weighted unless otherwise specified. The overall fidelity score was calculated as:

$$\text{Fidelity}\;\left(\%\right)=\left[\Sigma \left(\text{Itemi}\times \mathrm{wi}\right)/\mathrm{\Sigma wi}\right]\times 100$$

Where wi represents the assigned item weight and was set to 1 for all items in this assessment. Interpretation thresholds were defined a priori as low (<65%), moderate (65–80%), and high (>80%). Fidelity findings were interpreted alongside screening performance, index-testing cascade results, and qualitative findings to identify implementation constraints likely to affect consistency, uptake, and programme yield.

#### Integration of findings

Quantitative and qualitative findings were integrated during interpretation using the study objectives as an organizing framework. Screening-rule performance, index-testing cascade outcomes, healthcare-worker perspectives, and fidelity findings were compared to identify areas of convergence and divergence. This integration was used to assess how the targeted testing model functioned under routine implementation conditions, how implementation barriers may have influenced observed performance, and which programme components required refinement. Findings from the screening assessment informed interpretation of triage efficiency, cascade findings informed understanding of contact case-finding and linkage, and qualitative and fidelity findings helped explain implementation gaps affecting consistent delivery of risk screening, index testing, and HIV self-testing pathways.

### Ethics

Ethical approval was obtained from the Medical Research Council of Zimbabwe (MRCZ/A/2783) and the University of Zimbabwe Joint Research Ethics Committee (JREC/280/2021). Written informed consent was obtained from all qualitative interview participants. For the screening-rule and index-testing analyses, data were abstracted from routine programme sources and de-identified before analysis. No personal identifiers were included in the analytic datasets.

### Use of generative or machine-learning tools

During manuscript revision, a generative language tool was used to support language editing, organization, and clarification of selected passages. It was not used for study design, data collection, data management, statistical analysis, qualitative coding, generation of results, or independent interpretation of findings. All suggested revisions were critically reviewed and verified against the underlying study records and analyses by the authors, who take full responsibility for the accuracy and integrity of the manuscript.

## Results

### Screening tool development, item evaluation and assessment of the final combined rule

**Fig. 1:**
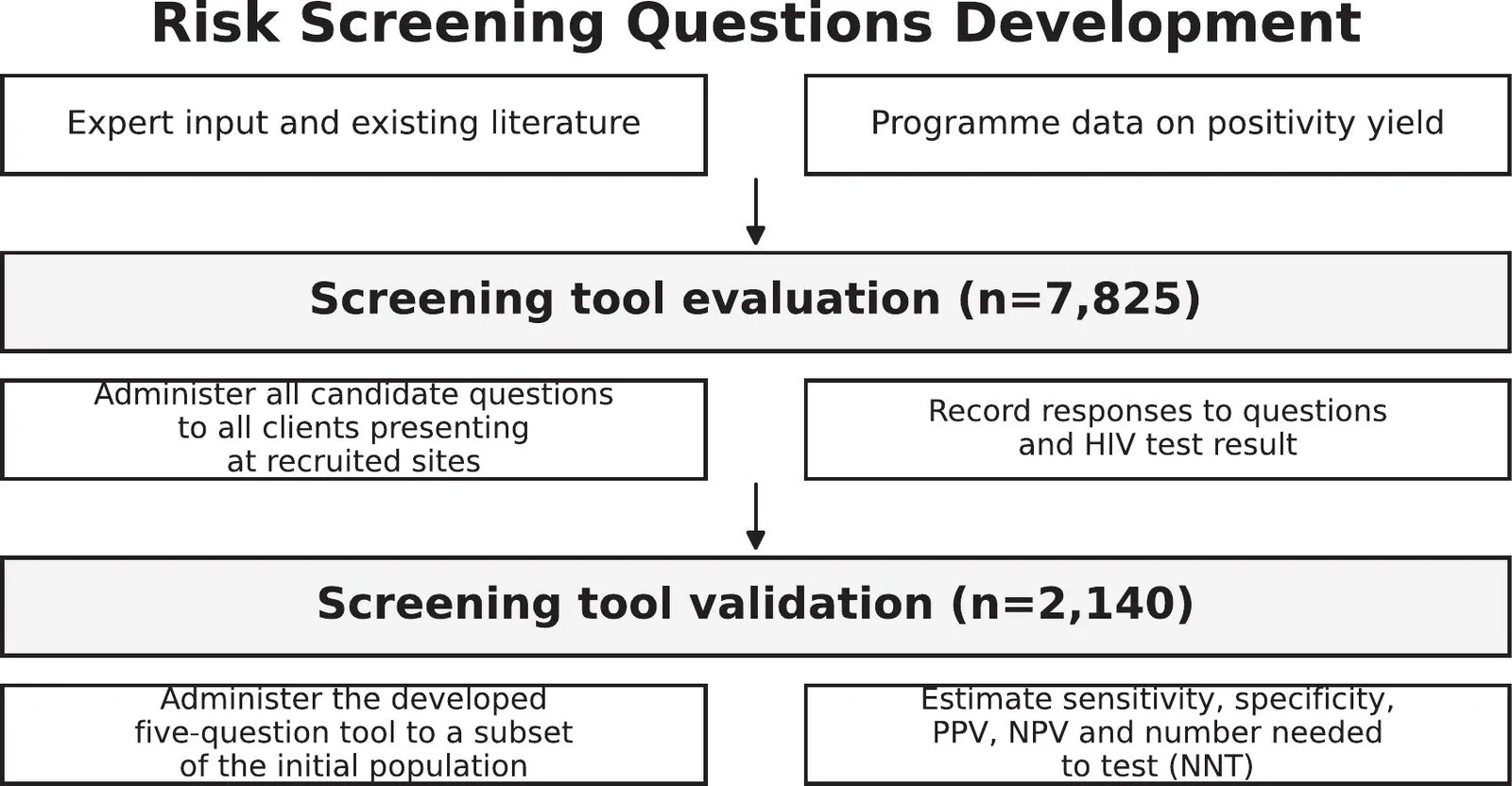
Development, initial item evaluation, and assessment of the final HIV risk-screening rule. Candidate screening questions were derived from programme data and existing literature and evaluated by administering the full 13-item set to clients attending recruited sites during the initial item-evaluation phase (*n* = 7825). The final combined screening rule was subsequently assessed among 2140 clients, with performance estimated in the complete-case subset (*n* = 2116). Performance measures included sensitivity, specificity, positive and negative predictive values, testing-volume reduction, and number needed to test.

The development, initial item evaluation, and subsequent assessment pathway for the HIV risk-screening tool is summarized in Fig. 1. Thirteen candidate screening questions were derived from programme experience, published evidence, and risk-profiling questions previously used locally or within the region. These questions were evaluated among 7825 clients attending 64 facilities during routine HIV testing service delivery. The full candidate question set is provided in Appendix B. The purpose of the initial phase was to identify a parsimonious set of domains that balanced screening performance with feasibility for routine service delivery. Following item evaluation and operational refinement, five domains were incorporated into the final screening tool reproduced in Appendix A.

During the initial item-evaluation phase, overall HIV positivity was 7.5% (584/7825). Item-level screening performance for the five retained questions is presented in Table 1, while raw counts for all 13 candidate questions are provided in Table S1. Self-perceived HIV risk had the highest sensitivity at 86.8% (95% CI 83.8–89.5), but low specificity at 26.1% (95% CI 25.1–27.1). Reporting that a sexual partner had tested HIV-positive in the previous 2 years had the next highest sensitivity at 68.3% (95% CI 64.4–72.1), followed by last HIV test at least 1 year previously or never tested at 63.4% (95% CI 59.3–67.3). Poor health and symptoms or signs of a sexually transmitted infection in the previous 12 months had lower sensitivity but higher specificity, suggesting that these items were more useful as part of a combined rule than as stand-alone screening questions.

**Table 1:** Sensitivity and specificity of the five retained screening questions in the initial item-evaluation phase (*N* = 7825).

Screening question	True positive (TP)	False negative (FN)	True negative (TN)	False positive (FP)	Sensitivity % (95% CI)	Specificity % (95% CI)
Self-perceived HIV risk	507	77	1874	5304	86.8 (83.8–89.5)	26.1 (25.1–27.1)
Partner HIV-positive in last 2 years	399	185	3767	3411	68.3 (64.4–72.1)	52.5 (51.3–53.6)
Last tested ≥1 year ago / never tested	370	214	3830	3348	63.4 (59.3–67.3)	53.4 (52.2–54.5)
Experienced poor health in past 12 months	246	338	5664	1514	42.1 (38.1–46.2)	78.9 (77.9–79.8)
Signs or symptoms of STI in past 12 months	240	344	5399	1779	41.1 (37.1–45.2)	75.2 (74.2–76.2)

TP = HIV-positive clients meeting the item-specific screening criterion; FN = HIV-positive clients not meeting the criterion; TN = HIV-negative clients not meeting the criterion; FP = HIV-negative clients meeting the criterion. The initial item-evaluation phase used a 12-month recall period for poor health and symptoms or signs of a sexually transmitted infection. The complete set of 13 candidate screening questions is provided in Appendix B.

The subsequent assessment phase enrolled 2140 clients, of whom 2116 contributed to complete-case analyses. Of the 2140 clients enrolled in the assessment of the final combined screening rule phase, 24 were excluded from complete-case analyses because of incomplete data and/or non-definitive HIV test results. The subsequent assessment phase enrolled 2140 clients. Of these, 24 were excluded because of incomplete screening data and/or non-definitive HIV test results, leaving 2116 clients in the complete-case assessment dataset. HIV positivity in this dataset was 7.98% (169/2116).

### Performance of individual screening questions and the final combined rule

Performance of the retained individual screening questions and the final combined rule in the complete-case assessment dataset is presented in Table 2. Individual items varied in their balance between sensitivity and specificity. Poor health in the previous 3 months had the highest positive predictive value (25.1%) and lowest number needed to test (NNT = 4), while reporting a known HIV-positive partner in the previous 2 years had a positive predictive value of 22.2% and NNT of 5. However, individual items alone had limited sensitivity. The final combined rule therefore prioritized sensitivity by screening in clients who had last tested at least 3 months previously, or had never tested, and reported at least one additional risk or symptom criterion.

**Table 2:** Performance of individual screening criteria and the final combined rule in the complete-case assessment sample, Zimbabwe, 2021 (*n* = 2116).

Screening question or criterion	TP	FN	TN	FP	Sensitivity, % (95% CI)	Specificity, % (95% CI)	PPV, % (95% CI)	NPV, % (95% CI)	NNT	Testing reduction, %
Last tested ≥12 months ago or never tested	92	77	1281	666	54.4 (46.6–62.1)	65.8 (63.6–67.9)	12.1 (9.9–14.7)	94.3 (93.0–95.5)	8	64
Self-perceived HIV risk	84	85	1373	574	49.7 (41.9–57.5)	70.5 (68.4–72.5)	12.8 (10.3–15.6)	94.2 (92.8–95.3)	8	69
Partner tested HIV-positive within the previous 2 years	36	133	1821	126	21.4 (15.5–28.4)	93.5 (92.3–94.6)	22.2 (16.1–29.4)	93.2 (92.0–94.3)	5	92
Poor health during the previous 3 months	53	116	1791	156	31.4 (24.5–38.9)	91.9 (90.6–93.0)	25.1 (19.4–31.5)	93.9 (92.7–94.9)	4	90
STI symptoms or signs during the previous 3 months	35	134	1752	195	20.7 (14.9–27.6)	90.0 (88.6–91.3)	15.2 (10.8–20.5)	92.9 (91.6–94.0)	7	89
Final combined screening rule^1^	**159**	**10**	**379**	**1568**	**94.1 (89.4–97.1)**	**19.5 (17.7–21.3)**	**9.2 (7.9–10.7)**	**97.4 (95.3–98.8)**	**11**	**18**

HIV positivity in the complete-case assessment sample was 8.0% (169/2116).

1 The final combined screening rule classified a client as screen-in for same-day provider-initiated HIV testing if the client had last tested for HIV at least 3 months previously, or had never tested, and reported at least one additional risk or symptom criterion. The assessment phase used a 3-month recall period for poor health and symptoms or signs of a sexually transmitted infection. The final operational tool is provided in Appendix A; the 13 candidate screening questions and five retained domains are presented in Appendix B. CI = confidence interval; TP = true positive; FN = false negative; TN = true negative; FP = false positive; PPV = positive predictive value; NPV = negative predictive value; NNT = number needed to test; STI = sexually transmitted infection.

The final combined rule achieved 94.1% sensitivity (95% CI 89.4–97.1), 19.5% specificity (95% CI 17.7–21.3), 9.2% positive predictive value, 97.4% negative predictive value, 18.0% testing-volume reduction, and NNT of 11. The rule identified 159 of 169 HIV-positive clients and missed 10 HIV-positive clients. It also classified 1568 HIV-negative clients as screen-in, indicating that the gain in sensitivity was accompanied by a substantial number of false-positive screens. This distinction between initial item-level performance in Table 1 and subsequent combined-rule performance in Table 2 is important because the final assessment used the refined implementation rule rather than the exact item wording used in the initial item-evaluation phase.

Because the final combined rule was designed as a triage tool for same-day provider-initiated testing, its low specificity should be interpreted in relation to the programme priority of minimizing missed HIV diagnoses rather than as failure to discriminate perfectly between HIV-positive and HIV-negative clients.

### Healthcare worker perspectives on targeted testing implementation

Twenty healthcare workers participated in the qualitative component; participant characteristics are summarized in Table S2. Participants described risk screening as feasible within routine workflows when staffing was adequate, screening tools were available, and criteria were clearly understood. Several healthcare workers reported that the tool could be incorporated into pre-test procedures without major disruption, particularly when job aids and standard operating procedures were available. One participant noted, “*It will be useful to have the tool as part of our guiding documents, it fits in very well.”*

Healthcare workers also described factors that limited consistent use of the screening rule. Reported barriers included staff shortages, competing service-delivery demands, overlapping documentation tools, limited confidential space, and variation in how screening criteria were interpreted. These barriers were perceived to reduce fidelity and increase the likelihood that clients would be screened inconsistently across providers or facilities. As one participant explained, *“The tool is good, but would be more useful when the workload doesn't suffocate us.”*

Index-testing implementation was described as high-yield but operationally demanding. Participants reported that successful contact elicitation and testing depended on adequate counselling time, client trust, confidentiality safeguards, and timely follow-up of elicited contacts. Healthcare workers described challenges in eliciting complete contact information, tracing contacts without unintended disclosure, and documenting follow-up outcomes across multiple registers or reporting tools. One participant stated, *“I have seen clients deliberately provide false information to misdirect us.”*

Participants identified several strategies that could strengthen the targeted testing model. These included simplified documentation tools, refresher training on the final screening rule and index-testing procedures, better availability of standard operating procedures and job aids, improved staffing support, and routine use of programme data during supervision. These suggestions were consistent with the fidelity findings, which identified gaps in SOP/job-aid use, staffing, screening tools, and documentation practices.

### Index testing cascade performance

**Fig. 2:**
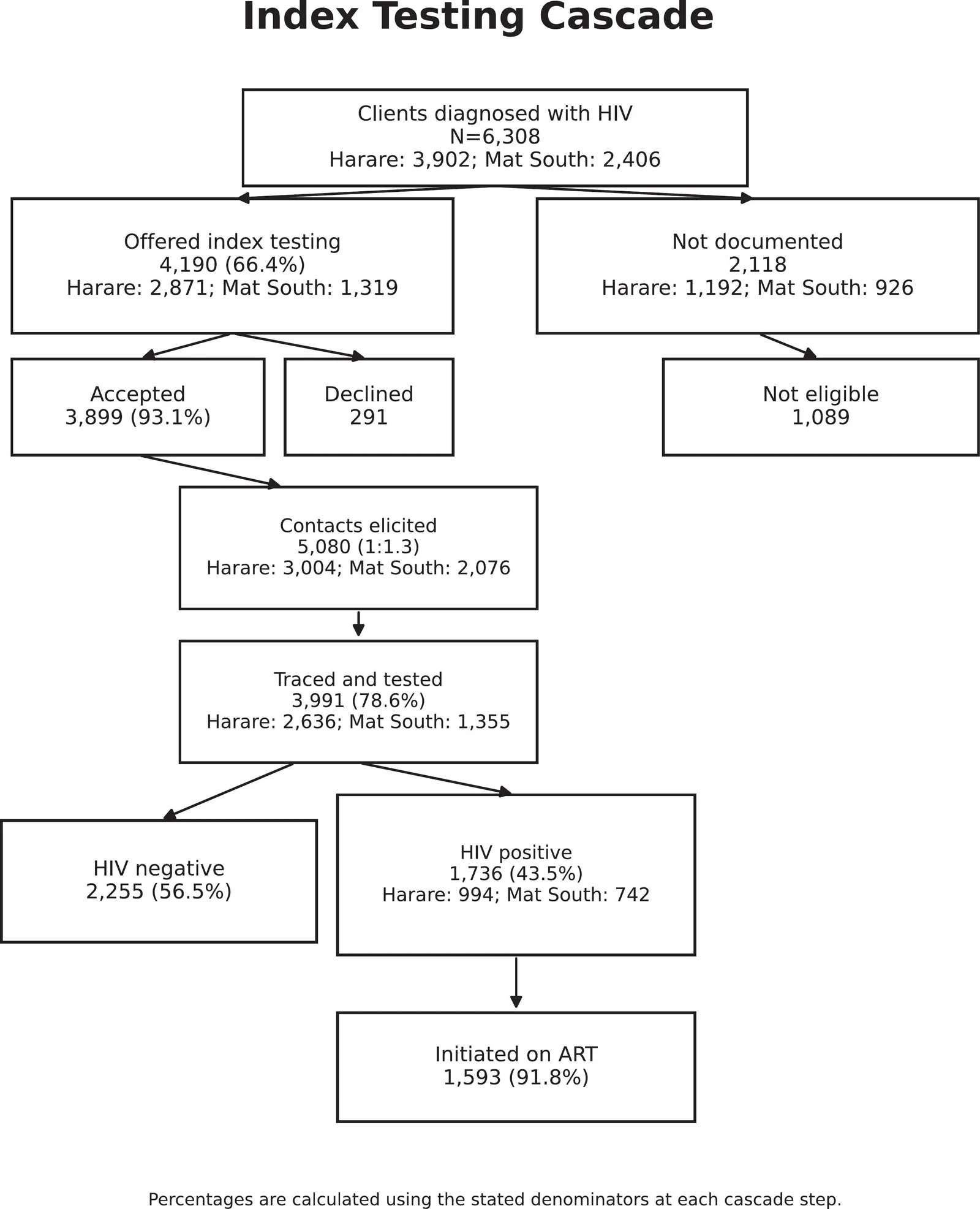
Index testing cascade among index clients in Harare and Matabeleland South, April 2021–June 2022 (*N* = 6308). The cascade summarizes documentation of index-testing offer, acceptance, contact elicitation, contact testing, HIV outcomes, and initiation of antiretroviral therapy (ART). Among contacts tested and receiving results (*n* = 3991), 1736 were HIV-positive (43.5%) and 2255 were HIV-negative (56.5%); 1593 (91.8%) of HIV-positive contacts-initiated ART. Percentages were calculated using the stated denominators at each cascade step.

The index-testing cascade is presented in Fig. 2. Among 6308 index cases included in the analysis, 4190 (66.4%) had documented offer of partner/index testing. Of those offered testing, 3899 (93.1%) accepted and 291 (6.9%) declined. A total of 5080 contacts were elicited, of whom 3991 (78.6%) were tested and had documented results.

Among contacts who were tested, 1736 (43.5%) were HIV-positive and 2255 (56.5%) were HIV-negative. Of the contacts testing HIV-positive, 1593 (91.8%) initiated antiretroviral therapy. These findings show that index testing was a high-yield component of the targeted testing model, while also demonstrating attrition at earlier cascade steps, particularly documentation of test offer, contact elicitation, and completion of contact testing.

### Factors associated with HIV positivity among index contacts

For tracing modality, anonymous contact tracing was compared with all other documented tracing approaches represented in the analytical dataset, which were combined as the reference category. Factors associated with HIV positivity among elicited contacts are shown in Table 3. In multivariable logistic regression, contact HIV positivity was higher among contacts traced through anonymous tracing (adjusted odds ratio [aOR] 8.46; 95% CI 3.37–22.75), those tested within 7 days of elicitation (aOR 2.78; 95% CI 2.44–3.18), male contacts (aOR 3.09; 95% CI 2.74–3.49), and first-time HIV testers (aOR 1.65; 95% CI 1.43–1.91). Spousal partners had lower odds of HIV positivity than regular partners (aOR 0.63; 95% CI 0.50–0.78). Because HIV positivity among tested contacts was common, these odds ratios should be interpreted as measures of association rather than approximations of risk ratios.

**Table 3:** Factors associated with HIV positivity among index contacts: category-specific frequencies and logistic regression estimates.

Factor	Category	HIV-positive, n/N (%)	HIV-negative, n/N (%)	Crude OR (95% CI)	Adjusted OR (95% CI)
Sex	Female	638/2787 (22.9)	2149/2787 (77.1)	ref.	ref.
	Male	1098/2293 (47.9)	1195/2293 (52.1)	3.09 (2.74–3.49)	3.09 (2.74–3.49)
Tracing modality	Other documented tracing modalities^1^	376/2092 (18.0)	1716/2092 (82.0)	ref.	ref.
	Anonymous contact tracing	13/20 (65.0)	7/20 (35.0)	8.48 (3.36–21.39)	8.46 (3.37–22.75)
HIV testing history	Previously tested	1256/3965 (31.7)	2709/3965 (68.3)	ref.	ref.
	First-time HIV test	443/1020 (43.4)	577/1020 (56.6)	1.66 (1.44–1.91)	1.65 (1.43–1.91)
Time from contact elicitation to testing	>7 days	530/2012 (26.3)	1482/2012 (73.7)	ref.	ref.
	≤7 days	996/1996 (49.9)	1000/1996 (50.1)	2.79 (2.44–3.18)	2.78 (2.44–3.18)
Relationship to index client	Regular partner	145/568 (25.5)	423/568 (74.5)	ref.	ref.
	Spousal partner	436/2452 (17.8)	2016/2452 (82.2)	0.63 (0.51–0.78)	0.63 (0.50–0.78)

Reference categories are listed first within each factor and denoted by “ref.” no odds ratio is reported for a reference category

^1^ “Other documented tracing modalities” comprises the non-anonymous tracing approaches recorded in the analytical dataset and serves as the reference category for anonymous contact tracing.

The adjusted model included sex, relationship to the index client, tracing modality, time from contact elicitation to testing, and prior HIV testing history. Because HIV positivity among tested contacts was common, the odds ratios should be interpreted as measures of association rather than as approximations of risk or prevalence ratios. Factor-specific denominators are reported because data availability varied across factors. OR = odds ratio; CI = confidence interval.

Table 3 also presents the category-specific frequencies and crude odds ratios underlying these associations. HIV positivity was 1098/2293 (47.9%) among male contacts compared with 638/2787 (22.9%) among female contacts. Positivity was 996/1996 (49.9%) among contacts tested within 7 days compared with 530/2012 (26.3%) among those tested later. Anonymous tracing had the highest crude positivity proportion at 13/20 (65.0%), although the number of contacts in this category was small and the estimate should be interpreted cautiously.

### Programme fidelity and selected province-level programme outputs

Programme fidelity findings indicated that implementation gaps were concentrated in the practical supports required for consistent delivery of targeted testing. Fidelity was stronger where registers and routine reporting tools were available, but weaker where standard operating procedures, job aids, screening tools, staffing support, and harmonized documentation tools were inconsistently available or used. These gaps aligned with healthcare-worker perspectives, which emphasized that staffing constraints, documentation burden, limited confidential space, and variable access to job aids affected consistent application of both risk screening and index testing procedures.

Selected province-level outputs from the index-testing and HIV self-testing components of the targeted-testing model are presented in Table 4. These indicators are shown together because both modalities contributed to the broader differentiated testing pathway, although they represent distinct programme components and use different denominators. The index-testing indicators describe programme implementation, HIV positivity among tested contacts, and ART initiation among contacts with positive HIV test results. The HIV self-testing indicators describe kit distribution, result reporting, reactivity among reported results, and confirmatory testing after a reactive self-test. Outputs were available for Manicaland and Matabeleland South and are therefore presented as descriptive provincial indicators rather than national estimates. For tracing modality, anonymous contact tracing was compared with the other documented tracing approaches represented in the analytical dataset, which were combined as the reference category.

**Table 4:** Selected programme outputs across index-testing and HIV self-testing pathways in Manicaland and Matabeleland South, Zimbabwe, 2021.

Programme component and indicator	Manicaland	Matabeleland South
Index-testing outputs		
Facilities implementing index testing	62.2% (61/98)	81.8% (72/88)
HIV positivity among tested index contacts	52.1% (461/884)	62.8% (742/1181)
ART initiation among index contacts with positive HIV test results	95.7% (441/461)	98.1% (728/742)
HIV self-testing outputs		
HIVST kits distributed	24,000	23,868
HIVST results reported or shared	57.7% (13,848/24,000)	57.7% (13,772/23,868)
Reactive results among reported HIVST results	7.7% (1066/13,848)	6.2% (854/13,772)
Confirmatory testing among clients with reactive HIVST results	97.5% (1039/1066)	95.0% (811/854)

ART = antiretroviral therapy; HIVST = HIV self-testing.

Index-testing and HIV self-testing outputs are presented together because both modalities contributed to the broader targeted-testing pathway, although they represent distinct programme components and use different denominators.

For the index-testing indicators, HIV positivity was calculated among index contacts who were tested and had a documented HIV test result. ART initiation was calculated among index contacts with positive HIV test results.

For the HIVST indicators, result reporting was calculated using the number of kits distributed as the denominator; reactivity was calculated among reported HIVST results; and confirmatory testing was calculated among clients with reported reactive HIVST results.

These indicators were available only for Manicaland and Matabeleland South and should not be interpreted as national estimates. Where denominators were derived arithmetically from documented aggregate numerators and percentages, they were used for descriptive reporting only and not for screening-performance estimates or regression analyses.

Programme fidelity findings were derived from implementation checklists and are therefore reported separately from the province-level output indicators in Table 4. Fidelity was stronger where registers and routine reporting tools were available, but weaker where standard operating procedures, job aids, screening tools, staffing support, and harmonized documentation systems were inconsistently available or used. These gaps converged with healthcare-worker accounts of staffing constraints, documentation burden, limited confidential space, and variable access to implementation guidance.

### Final targeted testing model

**Fig. 3:**
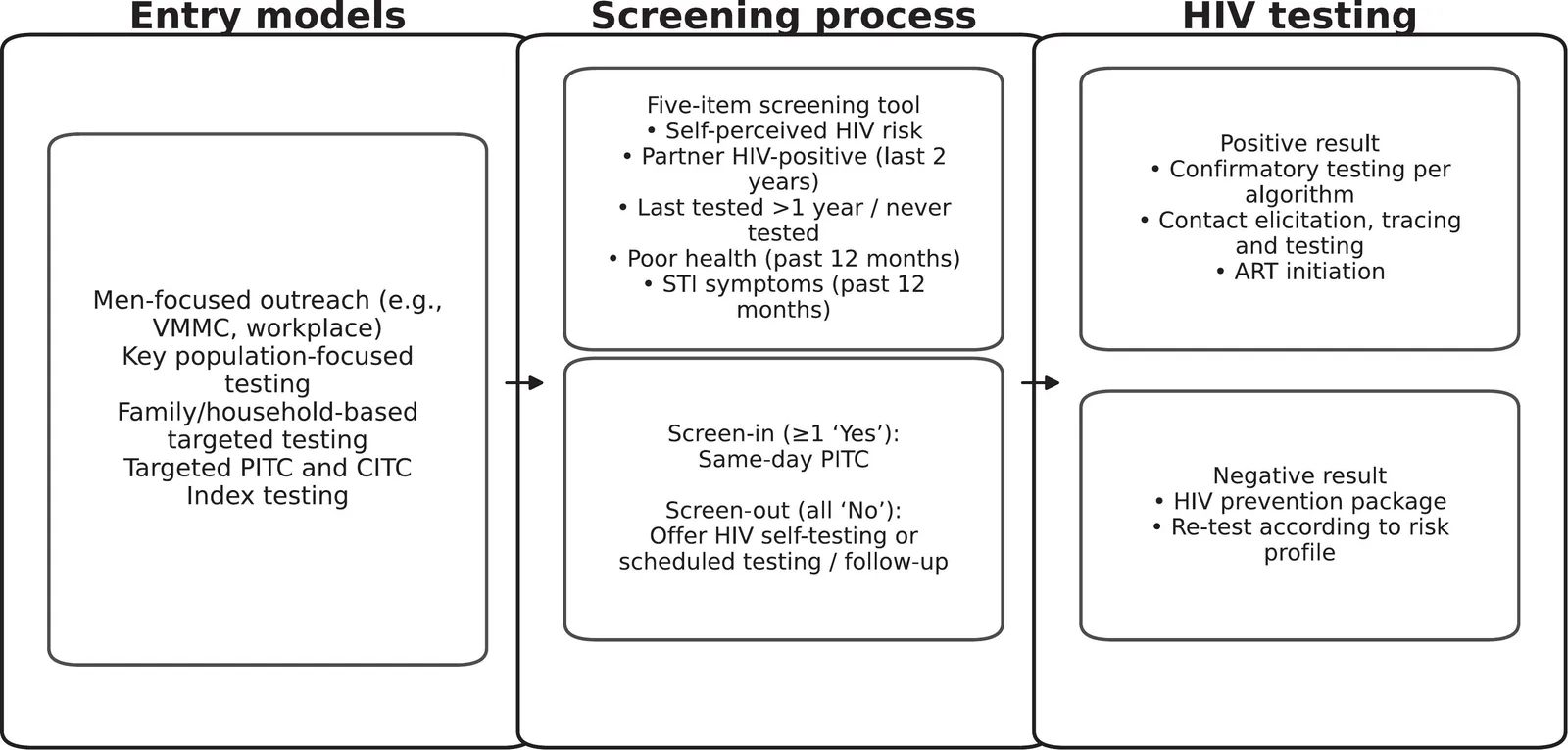
Final targeted HIV testing model for Zimbabwe. The model depicts routine service entry, application of the final combined screening rule, screening decision pathways, HIV testing options, index testing for clients with HIV-positive results, and linkage to treatment or prevention services. Under the final combined screening rule, clients are screened in for same-day provider-initiated HIV testing if they last tested for HIV at least 3 months previously, or have never tested, and report at least one additional risk or symptom criterion. Clients who screen negative are routed to HIV self-testing or scheduled/repeat testing according to programme procedures. Implementation supports include standard operating procedures, job aids, training, staffing, confidential counselling space, simplified documentation, and routine data review.

The final targeted HIV testing model is presented in Fig. 3. The model integrates evidence from the screening-rule assessment, index-testing cascade analysis, healthcare-worker perspectives, and programme fidelity assessment. It begins with clients presenting through routine service-delivery entry points, followed by application of the final combined screening rule. Clients are screened in for same-day provider-initiated HIV testing if they last tested at least 3 months previously, or have never tested, and report at least one additional risk or symptom criterion. Clients who screen negative are not excluded from testing; they are routed to HIV self-testing or scheduled/repeat testing according to programme procedures and client context. Clients with HIV-positive results are linked to antiretroviral therapy and index testing, while clients with HIV-negative results are linked to appropriate prevention services.

The model also emphasizes implementation supports, including standard operating procedures, job aids, training, staffing, confidential counselling space, simplified documentation, and routine data use.

## Discussion

### Principal findings

This mixed-methods implementation evaluation found that Zimbabwe's targeted HIV testing model, combining risk screening, index testing, HIV self-testing pathways, healthcare-worker implementation perspectives, and programme fidelity assessment, can support more efficient HIV case finding under routine programme conditions.

The final combined screening rule achieved high sensitivity (94.1%) and a high negative predictive value (97.4%), while reducing same-day provider-initiated testing volume by 18.0%. However, this gain in sensitivity occurred with low specificity (19.5%) and a modest positive predictive value (9.2%), indicating that the rule prioritized minimizing missed HIV diagnoses over minimizing false-positive screens.

In practical terms, the rule identified 159 of 169 HIV-positive clients but also classified 1568 HIV-negative clients as screen-in. The index-testing cascade demonstrated high yield among tested contacts, with 43.5% testing HIV-positive and 91.8% of those testing HIV-positive initiating antiretroviral therapy. These findings support index testing as an important high-yield component of the targeted testing model, while also showing attrition in documented offer, contact elicitation, and completion of contact testing. Healthcare-worker perspectives and fidelity findings highlighted the implementation supports needed to sustain the model, including clear standard operating procedures, job aids, adequate staffing, confidential counselling space, simplified documentation, and routine data use.

### Interpreting the screening trade-off

The final combined screening rule should be interpreted as a triage approach for same-day provider-initiated HIV testing rather than as a diagnostic discriminator. Its low specificity means that many clients classified as screen-in ultimately tested HIV-negative. This has practical implications for test-kit use, counsellor time, clinic flow, and documentation workload. However, in HIV case-finding programmes, a high-sensitivity rule may be appropriate when the priority is to reduce missed diagnoses, particularly when clients who screen negative are not denied testing but are routed to HIV self-testing or scheduled/repeat testing according to programme procedures.

The observed pattern of higher sensitivity with lower specificity is therefore not unexpected. It reflects the programme choice to accept more false-positive screens to preserve case detection. This trade-off should be considered when interpreting the 18.0% testing-volume reduction: the screening rule modestly reduced same-day testing volume, but it did not sharply discriminate between HIV-positive and HIV-negative clients. Future implementation should therefore monitor not only sensitivity and testing yield, but also provider workload, client acceptability, commodity use, linkage outcomes, and the performance of HIV self-testing or repeat-testing pathways for screen-negative clients.

### Comparison with existing evidence

The screening findings are consistent with evidence that brief risk-screening approaches can improve testing efficiency when they are embedded within routine clinical workflows and supported by clear implementation guidance [[9], [10], [11], [12]]. However, the findings also illustrate a common limitation of screening tools used in lower-prevalence or maturing epidemic contexts: high sensitivity may be achieved at the expense of specificity and positive predictive value. In this study, the final combined rule was useful primarily as a triage tool for prioritizing same-day provider-initiated HIV testing, rather than as a tool that sharply distinguished HIV-positive from HIV-negative clients [[11], [12]].

The individual screening items contributed differently to the final rule. Self-perceived risk and testing history supported broader case detection, whereas poor health, sexually transmitted infection symptoms, and known exposure to an HIV-positive partner were more specific but less sensitive as individual items. This pattern supports use of a combined rule rather than reliance on single screening questions. It also reinforces the importance of monitoring both missed diagnoses and false-positive screens when implementing targeted HIV testing approaches [13].

The index-testing findings are consistent with previous evidence that partner and family-based testing can achieve high positivity yield when contact elicitation, tracing, testing, and linkage are implemented with adequate counselling and confidentiality safeguards [[6], [8], [14], [15]]. The high positivity among tested contacts in this study supports index testing as a central component of the targeted testing model. At the same time, attrition before contact testing shows that high yield among those tested does not automatically translate into full programme impact unless offer, acceptance, elicitation, tracing, and documentation are consistently implemented [15].

### Implementation implications of the screening rule

The screening findings also have implementation implications. The final combined rule depends on consistent provider administration, correct interpretation of the testing-interval criterion, and reliable routing of screen-negative clients to alternative testing options. Because the rule has low specificity, inconsistent application could increase unnecessary same-day testing without preserving the intended sensitivity benefits. Conversely, overly restrictive interpretation could increase missed diagnoses.

These findings highlight the importance of standard operating procedures, job aids, refresher training, and routine monitoring of both screen-in and screen-negative pathways [[9], [10], [11], [12]].

### Implications on implementation fidelity

The qualitative and fidelity findings indicate that the effect of the targeted-testing model depends not only on the intrinsic performance of the screening rule or the observed yield of index testing, but also on the conditions under which these components are delivered. Clear operating procedures, accessible job aids, adequate staffing, confidential counselling space, streamlined documentation, and routine review of cascade data are therefore core implementation requirements. Without these supports, inconsistent screening may erode the intended sensitivity of the rule, while incomplete contact elicitation, delayed tracing, and fragmented documentation may reduce the population-level contribution of otherwise high-yield index testing [[14], [16]].

### Implications for model revision and policy uptake

The findings support a refined targeted HIV testing model in which risk screening, index testing, and HIV self-testing are implemented as complementary pathways rather than isolated modalities. The screening rule can help prioritize same-day provider-initiated HIV testing while maintaining alternative testing options for clients who screen negative. Index testing can then extend case-finding to sexual partners and biological children of HIV-positive clients, while HIV self-testing can provide an additional route for clients or contacts who are not immediately tested through facility-based services.

For policy and programme uptake, the screening rule should be positioned as a triage mechanism, not as a diagnostic test or a basis for denying testing. This distinction is important for maintaining client-centred access, preserving trust in HIV testing services, and reducing the risk that screen-negative clients are lost without alternative testing options. Implementation guidance should therefore specify how to administer the rule, how to route screen-negative clients, how to document screening outcomes, and how to monitor missed diagnoses, false-positive screens, and linkage outcomes.

The index-testing results also indicate that programme gains are likely to depend on timely tracing, confidentiality safeguards, and strong linkage systems. Contacts tested within 7 days and those reached through anonymous tracing had higher odds of HIV positivity, although odds ratios should be interpreted cautiously because contact positivity was common. Programme scale-up should therefore focus not only on expanding index-testing coverage but also on improving the quality, safety, timeliness, and documentation of contact services [17].

In this model, HIV self-testing strengthens differentiated access but requires clear procedures for result reporting, confirmatory testing after reactive self-tests, and linkage to treatment or prevention.

## Strengths and limitations

A major strength of this study is its comprehensive implementation-evaluation design, integrating screening-rule assessment, index-testing cascade analysis, qualitative inquiry, and programme fidelity assessment across diverse routine service-delivery contexts. The screening component included large evaluation and assessment samples, while the index-testing analysis drew on a substantial contact dataset. Reporting raw contingency-table counts, crude and adjusted measures of association, and explicit denominators improves transparency and reproducibility [[5], [15]].

The qualitative and fidelity components strengthened interpretation by identifying implementation barriers and supports that may influence whether targeted testing algorithms perform as intended in routine practice.

Several limitations should be considered. First, the screening tool changed between the initial item-evaluation phase and the subsequent assessment phase. The symptom recall period was shortened and the testing-interval criterion was refined before assessment of the final combined rule. Therefore, the October 2021 assessment should not be interpreted as validation of an unchanged instrument. Rather, it provides performance evidence for the final implementation rule; further external validation in additional settings would be needed to establish its performance more broadly.

Second, reliance on routine programme data introduces the possibility of incomplete documentation, misclassification, and variable data quality. Although standardized extraction, de-duplication, and supervisory review were used, residual error may persist. Third, facility and province selection was purposive and may limit generalizability to districts with different service-delivery models, staffing patterns, or HIV epidemiology. Fourth, qualitative interviews were conducted in selected provinces and may not capture the full national range of implementation experiences.8 9Fifth, logistic regression was used to examine factors associated with HIV positivity among index contacts.

Because HIV positivity among tested contacts was common, odds ratios may overstate the corresponding risk or prevalence ratios. These estimates should therefore be interpreted as measures of association rather than direct estimates of relative risk. Sixth, HIV self-testing outputs were available for selected provinces and should be interpreted as descriptive programme indicators rather than national HIVST estimates.

Finally, some province-level programme-output denominators were derived arithmetically from documented aggregate programme summaries when reports provided numerators and percentages rather than complete raw denominators. These derived values were used only for descriptive programme-output reporting and not for individual-level screening performance estimates or regression models. They should be interpreted cautiously unless verified against original programme registers. Future work should assess external validation of the final screening rule, costs, client acceptability, sustainability, and longer-term population-level effects as the targeted testing model is implemented more widely [9].

## Conclusion

In Zimbabwe's mature epidemic setting, a final combined HIV risk-screening rule integrated into routine HIV testing services maintained high sensitivity while modestly reducing same-day provider-initiated testing volume. The rule should be interpreted as a triage approach rather than a diagnostic discriminator, given its low specificity and substantial number of false-positive screens.

Index testing achieved high positivity among tested contacts and high linkage to antiretroviral therapy, particularly where contacts were tested promptly and tracing procedures were implemented effectively. Sustaining and scaling the targeted testing model will require consistent use of standard operating procedures, job aids, training, adequate staffing, confidential counselling space, simplified documentation, and routine data review. The model offers a pragmatic framework for optimizing HIV case finding while preserving alternative testing pathways, including HIV self-testing, for clients not prioritized for same-day provider-initiated testing.

### What is already known on this topic

- In mature HIV epidemics, routine testing positivity may decline, increasing the need for differentiated testing approaches that preserve case detection while improving efficiency.
- Risk screening and index testing can improve HIV case-finding yield, but their performance depends on clear eligibility criteria, provider adherence, confidentiality safeguards, and implementation fidelity.
- Fragmented tools, staffing constraints, inconsistent use of standard operating procedures, and documentation burden can reduce the effectiveness of targeted HIV testing models.

### What this study adds

- This study provides a mixed-methods implementation evaluation of Zimbabwe's targeted HIV testing model, integrating screening-rule assessment, index-testing cascade analysis, healthcare-worker perspectives, and programme fidelity assessment.
- The final combined screening rule maintained high sensitivity and reduced same-day provider-initiated testing volume by 18.0%, but low specificity indicates that it should be interpreted as a triage rule rather than a diagnostic discriminator.
- Index testing achieved high HIV positivity among tested contacts and strong linkage to antiretroviral therapy, while qualitative and fidelity findings identified practical supports needed for sustained implementation, including standard operating procedures, job aids, adequate staffing, confidential counselling space, simplified documentation, and routine data use.

### Implications for practice or policy

- Targeted HIV testing programmes should monitor both case detection and false-positive screens when implementing high-sensitivity risk-screening rules.
- Clients who screen negative should remain connected to alternative testing pathways, including HIV self-testing or scheduled/repeat testing.
- Programme scale-up should prioritize fidelity supports, including training, mentorship, job aids, confidential counselling space, simplified documentation, and routine cascade review.

## CRediT authorship contribution statement

**Hamufare Dumisani Mugauri:** Writing – review & editing, Writing – original draft, Visualization, Validation, Supervision, Software, Resources, Project administration, Methodology, Investigation, Funding acquisition, Formal analysis, Data curation, Conceptualization. **Owen Mugurungi:** Writing – review & editing, Visualization, Supervision, Resources, Methodology, Investigation, Data curation, Conceptualization. **Joconiah Chirenda:** Writing – review & editing, Visualization, Supervision, Methodology, Formal analysis, Data curation, Conceptualization. **Kudakwashe Takarinda:** Writing – review & editing, Validation, Software, Methodology, Investigation, Formal analysis, Data curation. **Mufuta Tshimanga:** Writing – review & editing, Validation, Supervision, Project administration, Methodology, Investigation, Conceptualization.

## Funding

This research did not receive any specific grant from funding agencies in the public, commercial, or not-for-profit sectors.

## Declaration of competing interest

The authors declare that they have no known competing financial interests or personal relationships that could have appeared to influence the work reported in this paper.

## Figures and Tables

**Table:** 

Field	Response
Province	______________________________
District	______________________________
Health facility	______________________________
Completed by	______________________________
Sequential number	______________________________
Date of screening, DD/MM/YYYY	____ / ____ / ________
Sex at birth	☐ Female ☐ Male
Age in completed years	__________ years

**Table:** 

No.	Screening domain	Response options	Operational guidance
1	Timing of the most recent HIV test	☐ Within the past 3 months ☐ Within the past 12 months ☐ More than 12 months ago ☐ Never tested	A previous HIV test at least 3 months ago, or never having tested, satisfies the testing-interval component of the final combined rule.
1a	Previous HIV test result	☐ Positive ☐ Negative ☐ Inconclusive	Apply the relevant national HIV testing or retesting procedure.
2	Self-perceived HIV risk	☐ Not at all ☐ Low ☐ Medium ☐ High	Any reported level of perceived risk satisfies this additional criterion when the testing-interval criterion is also met.
2a	Previous inconclusive result	☐ Not applicable ☐ Applicable	Refer to the national HIV testing or retesting algorithm.
2b	Current ART status among clients with a previously positive result	☐ Yes ☐ No ☐ Not applicable	Do not continue administering the risk-screening tool to a client with a previously confirmed positive result. If not receiving ART, refer for HIV care and treatment services.
3	Partner HIV-status criterion within the previous 2 years	☐ Yes ☐ No ☐ Unknown	A response meeting this risk criterion indicates eligibility when combined with the testing-interval criterion.
4	Poor health during the previous 3 months	☐ Yes ☐ No	Poor health may include relevant symptoms, recent illness, admission to hospital, or other conditions specified in the accompanying standard operating procedure.
5	Symptoms or signs of a sexually transmitted infection	☐ Yes ☐ No	Administer according to the symptom definitions provided in the accompanying standard operating procedure.

**Table:** 

Outcome	Selection
Met the final combined screening rule	☐
Did not meet the final combined screening rule	☐
Referred for HIV self-testing	☐
Advised to return for scheduled or repeat testing	☐
Return interval	______ days/months
Other action	__________________

**Table:** 

Decision	Response
Accepted the HIV test	☐ Yes ☐ No
HIV test offered	☐ Yes ☐ No
Additional referral/action	__________________

**Table:** 

No.	Candidate screening question or domain	Initial recall period	Retained in final tool
1	When was the last time you were tested for HIV?	Previous testing history	Yes
2	Do you consider yourself to be at risk of HIV infection?	Current self-perception	Yes
3	Concurrent-partnership risk question	Previous 12 months	No
4	Have you experienced poor health in the past year?	Previous 12 months	Yes, refined
5	Have you experienced recurrent skin rashes in the past year?	Previous 12 months	No
6	Have you experienced significant weight loss in the past year?	Previous 12 months	No
7	Have you had a persistent or recurrent cough in the past year?	Previous 12 months	No
8	Have you experienced symptoms or signs of a sexually transmitted infection in the past year?	Previous 12 months	Yes, refined
9	Partner HIV-status criterion within the previous 2 years	Previous 2 years	Yes
10	Have you shared sharp objects with another person in the past year?	Previous 12 months	No
11	Transactional-exposure risk question	Previous 12 months	No
12	Non-consensual-exposure risk question	Previous 2 years	No
13	Condomless-exposure or condom-failure risk question	Previous 12 months	No

**Table:** 

Retained domain	Initial item-evaluation specification	Final operational specification
Timing of the previous HIV test	More than 1 year previously or never tested	At least 3 months previously or never tested
Self-perceived HIV risk	Self-perceived risk of HIV infection	Any reported level of perceived risk
Partner HIV status	Partner tested HIV-positive within the previous 2 years	Retained without change to the recall period
Poor health	Poor health during the previous 12 months	Poor health during the previous 3 months
STI symptoms or signs	Symptoms or signs during the previous 12 months	Symptoms or signs during the previous 3 months

## Data Availability

De-identified aggregate data that support the findings of this study are available from the corresponding author upon reasonable request and subject to Ministry of Health and Child Care approvals and applicable data-sharing agreements. The data are not publicly available because they were derived from routine programme sources under Ministry custodianship.

## Appendix A Appendices

Final Adult HIV Testing Services Risk-Screening Tool Operational version dated 11 March 2022.

### A.3 Final combined screening rule

A client meets the final combined screening rule for same-day provider-initiated HIV testing when both conditions apply:

• The client last tested for HIV at least 3 months previously or has never tested.
• The client meets at least one additional criterion relating to self-perceived HIV risk, partner HIV status, poor health during the previous 3 months, or symptoms or signs of a sexually transmitted infection.
• Clients who do not meet the final combined screening rule should not be categorically denied HIV testing. Depending on programme procedures, individual circumstances, client preference, and service-delivery capacity, they may be offered HIV self-testing or advised to undergo scheduled or repeat testing.

## Appendix B Candidate Screening Questions Evaluated and Domains Retained in the Final Tool

### B.1 Developmental context

During the initial item-evaluation phase, 13 candidate screening questions were administered to clients presenting for HIV testing services. Each question was evaluated against the client's definitive HIV test result. Item retention was informed by screening performance and operational feasibility, to identify a concise set of domains that maintained high sensitivity while remaining practical for routine service delivery.

### B.3 Five domains retained after initial item evaluation

• Timing of the previous HIV test
• Self-perceived HIV risk
• HIV status of a partner within the previous 2 years
• Poor health
• Symptoms or signs of a sexually transmitted infection

### B.4 Refinements before assessment of the final combined rule

Before the subsequent field assessment, the retained domains were operationally refined to improve clarity and standardize provider interpretation.

The testing-interval criterion was changed from more than 1 year to at least 3 months, while the recall periods for poor health and sexually transmitted infection symptoms or signs were shortened from 12 months to 3 months. These refinements were intended to reduce recall ambiguity and standardize administration under routine programme conditions.

### B.5 Final operational decision rule

Under the final combined rule, a client was classified as meeting the criterion for same-day provider-initiated HIV testing when:

• the client had last tested for HIV at least 3 months previously or had never tested; and
• the client met at least one additional retained risk or symptom criterion.
• The October 2021 phase therefore assessed the performance of the final combined implementation rule rather than validating an unchanged version of the original 13-item instrument.
