# Supplementary material for: Targeted HIV testing modalities in Zimbabwe: Achieving effectiveness through a revised model
Source: Glob Epidemiol. 2026 Sep 6;12:100288. doi: 10.1016/j.gloepi.2026.100288 (PMC13579930; doi:10.1016/j.gloepi.2026.100288)
Supplement: Supplementary file 1 [file mmc1.docx]

**Table S1. Full 13-item screening-question evaluation table with raw counts (N = 7,825)**

| **Screening question** | **TP** | **FN** | **TN** | **FP** | **Sensitivity % (95% CI)** | **Specificity % (95% CI)** |
| --- | --- | --- | --- | --- | --- | --- |
| Last tested ≥1 year ago | 370 | 214 | 3830 | 3348 | 63.4 (59.3–67.3) | 53.4 (52.2–54.5) |
| Self-perceived HIV risk (Yes vs No risk) | 507 | 77 | 1874 | 5304 | 86.8 (83.8–89.5) | 26.1 (25.1–27.1) |
| >1 concurrent sexual partner in past 1 year | 105 | 479 | 6596 | 582 | 18.0 (14.9–21.3) | 91.9 (91.2–92.5) |
| Experienced poor health in past 1 year | 246 | 338 | 5664 | 1514 | 42.1 (38.1–46.2) | 78.9 (77.9–79.8) |
| Recurrent skin rashes in past 1 year | 41 | 543 | 6980 | 198 | 7.0 (5.1–9.4) | 97.2 (96.8–97.6) |
| Significant weight loss in past 1 year | 135 | 449 | 6650 | 528 | 23.1 (19.8–26.8) | 92.6 (92.0–93.2) |
| Persistent/recurrent cough in past 1 year | 81 | 503 | 6854 | 321 | 13.9 (11.2–16.9) | 95.5 (95.0–96.1) |
| Symptoms/signs of STI in past 1 year | 240 | 344 | 5399 | 1779 | 41.1 (37.1–45.2) | 75.2 (74.2–76.2) |
| Sexual partner HIV-positive in last 2 years | 399 | 185 | 3767 | 3411 | 68.3 (64.4–72.1) | 52.5 (51.3–53.6) |
| Shared sharp objects in past 1 year | 23 | 561 | 7041 | 137 | 3.9 (2.5–5.9) | 98.1 (97.7–98.4) |
| Engaged in transactional sex in past 1 year | 100 | 484 | 6514 | 664 | 17.1 (14.2–20.4) | 90.7 (90.1–91.4) |
| Forced sex in past 2 years | 35 | 546 | 6942 | 236 | 6.5 (4.6–8.8) | 96.7 (96.3–97.1) |
| Sex without condom with unusual partner / condom burst | 87 | 497 | 6557 | 621 | 14.9 (12.1–18.0) | 91.3 (90.7–92.0) |

*TP = true positive; FN = false negative; TN = true negative; FP = false positive. Evaluation-phase wording used a 12-month recall period for poor health and STI symptoms.*
