# Supplementary material for: Targeted HIV testing modalities in Zimbabwe: Achieving effectiveness through a revised model
Source: Glob Epidemiol. 2026 Sep 6;12:100288. doi: 10.1016/j.gloepi.2026.100288 (PMC13579930; doi:10.1016/j.gloepi.2026.100288)
Supplement: Supplementary file 2 [file mmc2.docx]

**Table S2. Characteristics of healthcare workers participating in the qualitative component (N = 20)**

| **Characteristic** | **Category** | **n (%)** |
| --- | --- | --- |
| Gender | Female | 13 (65%) |
|  | Male | 7 (35%) |
| Cadre | Primary counsellor | 9 (45%) |
|  | Charge nurse / sister-in-charge | 6 (30%) |
|  | Matron | 1 (5%) |
|  | Other (Doctor / RGN / PCN) | 4 (20%) |
| Years at current facility | <2 years | 9 (45%) |
|  | 2–5 years | 8 (40%) |
|  | >5 years | 3 (15%) |

*Participant characteristics are presented in supplementary material to keep the main Results focused on implementation themes rather than descriptive demographics alone.*
